# Circadian Phenotype Composition is a Major Predictor of Diurnal Physical Performance in Teams

**DOI:** 10.3389/fneur.2015.00208

**Published:** 2015-10-01

**Authors:** Elise Facer-Childs, Roland Brandstaetter

**Affiliations:** ^1^School of Biosciences, University of Birmingham, Birmingham, UK

**Keywords:** circadian, physical, mental, performance, sleep

## Abstract

Team performance is a complex phenomenon involving numerous influencing factors including physiology, psychology, and management. Biological rhythms and the impact of circadian phenotype have not been studied for their contribution to this array of factors so far despite our knowledge of the circadian regulation of key physiological processes involved in physical and mental performance. This study involved 216 individuals from 12 different teams who were categorized into circadian phenotypes using the novel RBUB chronometric test. The composition of circadian phenotypes within each team was used to model predicted daily team performance profiles based on physical performance tests. Our results show that the composition of circadian phenotypes within teams is variable and unpredictable. Predicted physical peak performance ranged from 1:52 to 8:59 p.m. with performance levels fluctuating by up to 14.88% over the course of the day. The major predictor for peak performance time in the course of a day in a team is the occurrence of late circadian phenotypes. We conclude that circadian phenotype is a performance indicator in teams that allows new insight and a better understanding of team performance variation in the course of a day as often observed in different groupings of individuals.

## Introduction

Whether it is in the sports world, the academic world, or the corporate world, the pressures on personal best and team performances in modern society are growing and understanding the influencing factors affecting optimal performance, which span from cognitive and physical abilities to expert skills, training, and experience (1–3), becomes increasingly important. Both physical and mental performances are of great significance to individuals and teams when trying to maximize productivity or optimize performance and positive links between physical and mental performance have been identified in a considerable number of studies (4–11). Various factors are involved in overall performance, including intensity of activity, duration, response time (12, 13), the effect of physical activity on executive cognitive function (13–18), and individual fitness, which have impacts on post-exercise mental performance (19). Critical decision-making is also imperative to overall optimal performance. Macora and colleagues (20) described that a state of “mental fatigue” occurs after extended periods of cognitive processing and found that exercise tolerance could be affected by the state of mental fatigue, impairing physical performance. Interestingly, it was the perception of effort that was significantly different under a mental fatigued state, and not any physiological functions, such as cardiovascular mechanisms (20). It has, therefore, been proposed in the motivation intensity theory that perceived exertion and potential motivation influence performance (21). Further interactions between circadian processes and sleep homeostasis in human performance have been described in the two-process model of sleep regulation (22). Disturbances in these processes have been shown to affect neural activation and brain metabolism, ultimately influencing mental and physical performance (23). This model has been adapted to account for a buildup of sleep debt by McCauley and colleagues (24). In this model, the effect on performance is determined by the daily time spent awake and a recent study showed that physical performance strongly depends on time since awakening (25) supporting the view that this model requires further development and consideration of further sleep related parameters (23).

A complex network of endogenously driven biological clocks regulates virtually all physiological and behavioral diurnal variations in humans. Circadian rhythmicity has been shown to contribute to the regulation of key physiological and cognitive processes involved in performance, including plasma levels of hormones, glucose tolerance, core body temperature, blood pressure, and performance variables, such as reaction times, alertness, and memory speed (26–30). The master circadian oscillator, the hypothalamic suprachiasmatic nucleus (SCN), acts as an internal coordinator and synchronizer at the whole-organism level (31–33). At the cellular level, circadian rhythm generation is based on interlocking molecular feedback loops and post-translational modifications, referred to as the transcriptional translational feedback loop (TTFL) (34). Individual circadian rhythms, i.e., whether individuals are “larks” or “owls” (35, 36), have strong impact on the individual performance (25). The differences between larks and owls, also called “morning/evening types” or “chronotypes” (35, 37), and referred to as circadian phenotypes (25) in this paper, are due to environmental influences, genetic variation, age, and gender. The combination of these factors results in disparities between individuals’ biological clocks and how they entrain to exogenous (environmental) cues, such as the environmental light/dark cycle and social factors (38).

In this paper, we are exploring the possible impact of individual diurnal performance variation in interdependent group situations (e.g., sports teams) depending on the within-group composition of circadian phenotypes. We use a simple model considering individual diurnal performance variation of different circadian phenotypes to predict team performance variability establishing circadian phenotype as an important performance indicator in teams.

## Experimental Procedures

### Participants and chronometric testing

Two hundred and sixteen individuals (114 females, 102 males, ages ranged from 16 to 35, average age of 21.5 ± 3.96 years), across 12 sports teams including 5 field hockey teams and 7 football teams (Table 1) with standards ranging from regional club to international level participated in this study. Participants were recruited during training sessions and all team members asked to complete the RBUB chronometric test. In 9 out the 12 teams, 100% of team members returned the completed chronometric test (teams 1, 3, 4, 5, 6, 7, 8, 11, and 12); in the remaining three teams, 82% (team 2), 91% (team 9), and 73% (team 10) of team members returned the completed chronometric test.

**Table 1:** **Details of composition, gender, total number of team players, and sport for each team**.

Team number	Male/female (M/F)	Sport	Number of participating team members (*N*)
T1	M	Football	17
T2	M	Football	9
T3	M	Football	30
T4	F	Football	32
T5	F	Field hockey	25
T6	F	Field hockey	30
T7	M	Field hockey	22
T8	F	Football	14
T9	M	Field hockey	10
T10	F	Field hockey	8
T11	F	Football	14
T12	M	Football	14

*The teams studied included a total of 216 competition-level athletes across 12 different sports teams. There were six male teams and six female teams. Individual ages ranged from 16 to 35, average age being 21.5 with a SD of 3.96*.

The chronometric questionnaire (RBUB chronometric test) (25) was developed to study sleep/wake-related circadian parameters as compared to training, competition, and performance variables. All data were collected according to the Human Ethics regulations of the University of Birmingham and all participants were informed that data collected were treated anonymously and held according to the Data protection Act 1998. The RBUB chronometric test (25) collects information on wake-up times, sleep-onset times, sleep-onset delays, sleep duration, alarm use, light exposure, food intake, exogenous schedules (work, training, competition, school and/or university timetables), sleep quality, daytime naps, periods of mental and physical high and low activity, energy drink consumption, alcohol consumption, caffeine consumption, and smoking. Completion of the chronometric test took athletes 10 min on average. For each individual, scores were allocated to wake-up times and sleep-onset times during weekdays, weekends, and free days, the time lag between weekday and weekend wake-up times, self-reported times of high (mentally and physically active) and low (tiredness, fatigue) activity periods and meal times. Masking factors, such as working hours, university timetables, and training schedules, were considered when allocating scores. Scores represented time in hours and were used to categorize into early (ECT), intermediate (ICT), and late (LCT) circadian phenotypes.

### Modeling of predicted physical performance rhythms

Predicted physical performance was calculated from previously collected performance-test results (25). Briefly, performance data from three different performance tests (BLEEP tests, sprints, and skills/accuracy tests) carried out at six different times of day between 07.00 and 22.00 h were analyzed separately for athletes of the three different circadian phenotypes, i.e., early (ECT), intermediate (ICT), and late (LCT), and diurnal performance curves produced for each circadian phenotype showing significantly different curve shapes and peak performance times in the course of a day (25).

From the previously obtained BLEEP test results (25), representative daily performance curves were generated for each circadian phenotype by using second-order polynomial non-linear regressions generating 15-min time interval curve fits. Peak performance times were determined as time of day of maximum values of second-order polynomial non-linear regression curves and peak performance values as percentage of maximum performance. The *x*/*y* data of these curve fits were then combined by averaging ECT, ICT, and LCT data to generate performance curves modeling predicted diurnal team performance curves according to the distribution of circadian phenotypes for each team. Second-order polynomial non-linear regressions were used as curve fits throughout the study to calculate performance values as a function of time of day as well as to determine peak performance values. Kruskal–Wallis test was used to test differences of circadian and performance parameters between circadian phenotypes between teams and between different times of day for statistical significance. Dunn’s multiple comparison test was used to compare individual group mean values.

## Results

### Circadian phenotyping

**Figure 1:**
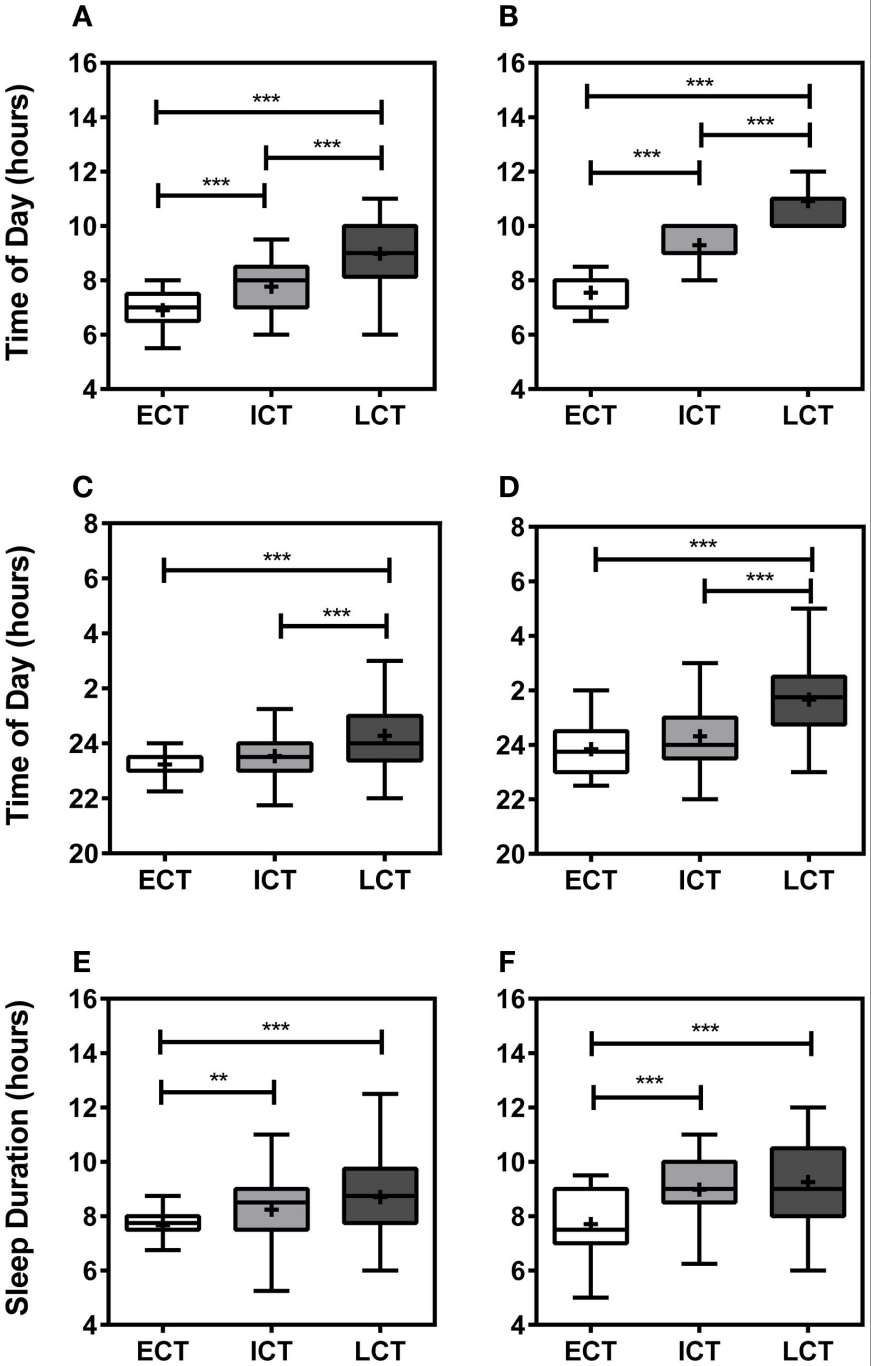
**Relevant sleep/wake parameters validate circadian phenotyping**. **(A)** Average wake-up time on weekdays. **(B)** Average wake-up time at weekends. **(C)** Average sleep onset on weekdays. **(D)** Average sleep onset at weekends. **(E)** Average sleep duration during weekdays. **(F)** Average sleep duration weekends. White boxes represent early circadian phenotypes (ECT), light gray boxes represent intermediate circadian phenotypes (ICT), late circadian phenotypes are shown in dark gray (LCT). Boxplots show 25th–75th percentile. Whiskers and outliers are plotted by the Tukey method and the mean is shown within the box as a +. Statistical analysis was carried out using Kruskal–Wallis non-parametric tests combined with Dunn’s multiple comparison test. ns, not significant, **p < 0.01, ***p < 0.001.

Following comprehensive analysis and consideration of specific sleep/wake-related criteria, including wake-up times on weekdays, weekends, and training-free days, sleep onset on weekdays, weekends, and training-free days, sleep durations, periods of high and low activity, sleep inertia, and meal times, as previously described (25), all participants could be categorized into “circadian phenotypes” (CT). In total, we identified 15% early circadian phenotypes (ECT; *n* = 32), 51% intermediate circadian phenotypes (ICT; *n* = 111), and 34% late circadian phenotypes (LCT; *n* = 73). The circadian phenotyping methodology proved consistent with relevant circadian parameters, such as wake-up times, sleep-onset times, and sleep durations (Figure 1). Average wake-up times differed significantly between circadian phenotypes (Kruskal–Wallis, *p* < 0.001) being 6.90 ± 0.11 h for ECTs on weekdays as compared to 7.55 ± 0.11 h on weekends, for ICTs 7.77 ± 0.08 h at weekdays and 9.29 ± 0.06 h on weekends, and for LCTs 8.98 ± 0.14 h on weekdays and 10.91 ± 0.10 h on weekends (Figures 1A,B). Significant differences were also seen in sleep-onset times (Kruskal–Wallis, *p* < 0.001) and sleep duration times (Kruskal–Wallis, *p* < 0.001). ECT sleep onset was 23.23 ± 0.13 h on weekdays and 23.84 ± 0.17 h on weekends, whilst ICT sleep onset was 23.53 ± 0.08 h on weekdays and 24.32 ± 0.10 h on weekends. LCT average sleep onset was 24.27 ± 0.13 h on weekdays and 01.66 ± 0.16 h on weekends (Figures 1C,D). ECTs slept for 7.66 ± 0.12 h on weekdays and 7.70 ± 0.22 h on weekends, ICTs for an average of 8.24 ± 0.11 h on weekdays and 8.97 ± 0.12 h on weekends, and LCTs for 8.70 ± 0.17 h on weekdays and 9.25 ± 0.17 h on weekends (Figures 1E,F).

### Team distribution of circadian phenotypes and predicted diurnal physical performance

**Figure 2:**
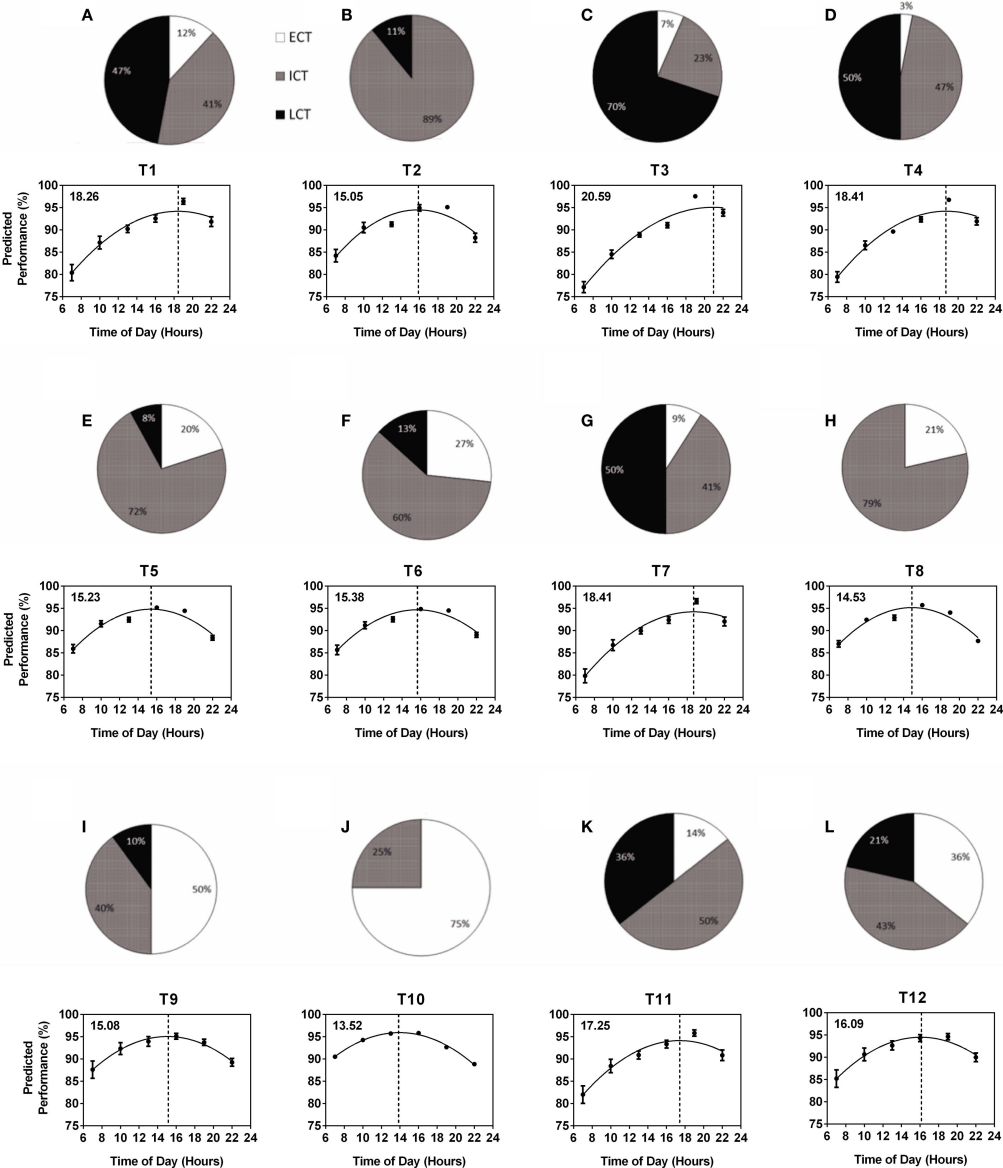
**Variability of within-team circadian phenotype composition determines predicted diurnal performance curves and peak performance times**. Each team (T1–T12) is represented by a pie chart showing the composition of circadian phenotypes and a graph showing predicted performance over the course of a day calculated from performance tests conducted at six different times of day (25). Peak performance times are indicated by the dotted vertical lines and shown in the top left hand corner of each graph. Early circadian phenotypes (ECT) are shown in white, intermediate circadian phenotypes (ICT) in gray and late circadian phenotypes (LCT) in black. **(A)** T1. **(B)** T2. **(C)** T3. **(D)** T4. **(E)** T5. **(F)** T6. **(G)** T7. **(H)** T8. **(I)** T9. **(J)** T10. **(K)** T11. **(L)** T12.

The composition of circadian phenotypes was highly variable between the 12 teams (T1–T12). The most prominent differences in circadian phenotype composition were found between one female team (T10) made up of 75% ECTs and 25% ICTs (average age 27.0 years) and one male team (T3) made up of 7% ECTs, 23% ICTs, and 70% LCTs (average age 19.9 years) (Figure 2). Overall, ECTs ranged from 0 (T2) to 75% (T10), ICTs from 23 (T3) to 89% (T2), and LCTs from 0 (T8, T10) to 70% (T3) (Figures 2A–L). Predicted diurnal peak performance times varied by 7.12 h between teams, with earliest peak performance found in T10 at 13.52 h and latest peak performance found in T3 at 20.59 h (Figures 2A–L). Several teams showed comparable performance peaks in the afternoon (T8 at 14.53 h, T2 at 15.05 h, T9 at 15.08 h, T5 at 15.23 h, and T6 at 15.38 h), while others showed their performance peaks significantly later, i.e., early evening (T1 at 18.26 h, T4 and T7 at 18.41 h) (Figures 2A–L). Further analysis showed a highly significant correlation between the number of LCTs and predicted team peak performance time with peak performance time being later in the day with increasing numbers of LCTs (Spearman non-parametric correlation, *p* < 0.001), while there was no significant correlation between the number of ECTs and ICTs and predicted team peak performance time (*p* = 0.06 and *p* = 0.25, respectively).

### Diurnal performance variation within and between teams

**Figure 3:**
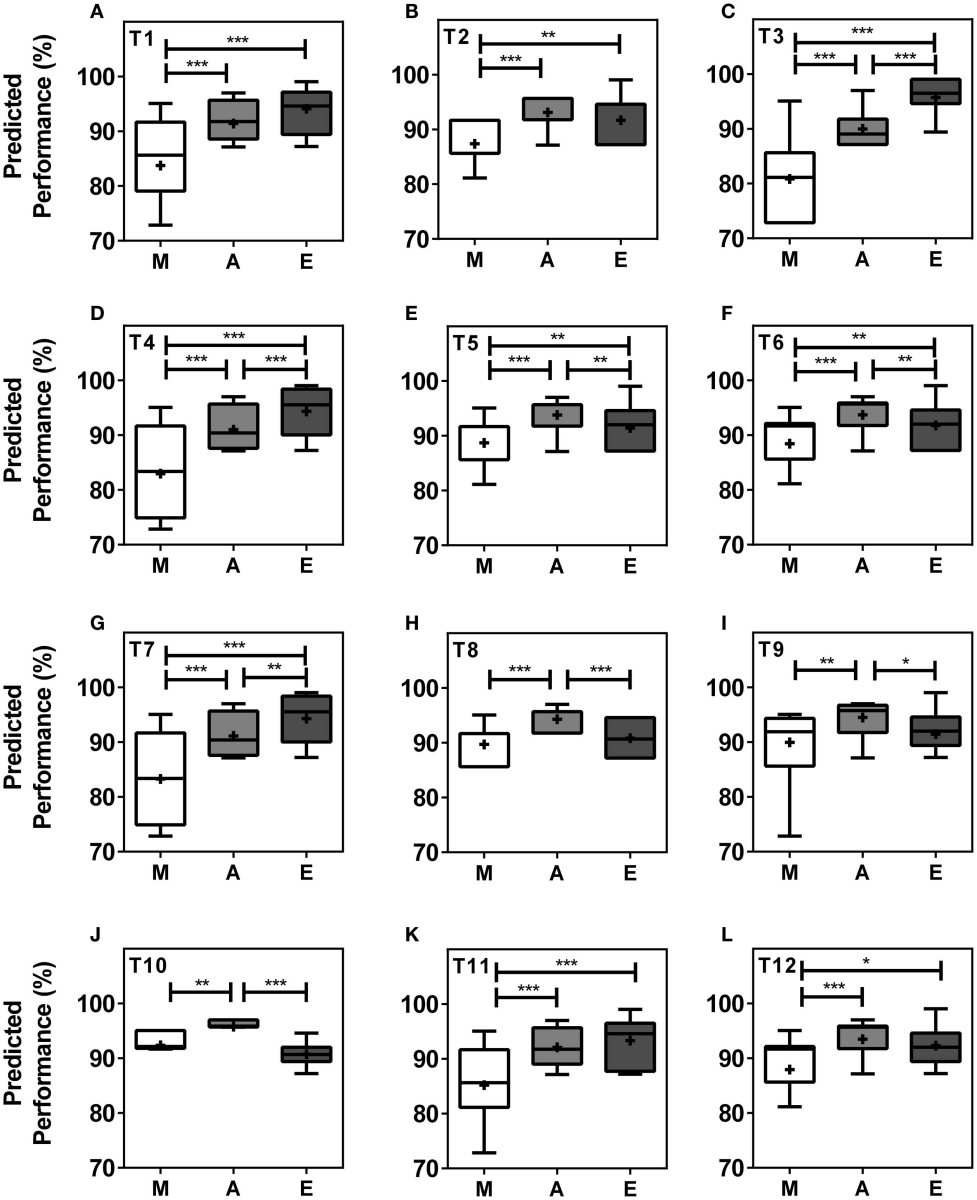
**Team performance undergoes significant changes in the course of a day within teams**. Boxplots represent predicted team performance levels of T1 **(A)** to T12 **(L)**. (*M*) = morning, i.e., 07.00–10.00 h, light gray bars; (*A*) = afternoon, i.e., 13.00–16.00 h, dark gray bars; (*E*) = evening, i.e., 19.00–22.00 h, black bars. Tukey boxplots show 25th–75th percentile; mean values are shown within the box as a +. Statistical analysis was carried out using Kruskal–Wallis non-parametric tests combined with Dunn’s multiple comparison test. *p < 0.05, **p < 0.01, ***p < 0.001.

When analyzing performance during morning (*M* = 07.00–10.00 h), afternoon (*A* = 13.00–16.00 h), and evening (*E* = 19.00–22.00 h) within teams, our model predicts significant differences in performance levels between at least two of the three different times of day in all teams and significant differences between all three times of day in five (42%) of the teams. In seven (58%) of the teams, performance values were highest in the afternoon, while performance levels peaked in the evening in five teams (Figures 3A–L). T3, T4, T5, T6, and T7 showed significant differences in peak performance between *M*/*A*, *A*/*E*, and *M*/*E* (Kruskal–Wallis, *p* < 0.001). T1, T2, T11, and T12 showed significant differences between *M*/*A* and *M*/*E* whilst significant differences were seen between *M*/*A*, between *A*/*E* in T10, and between *M*/*A* in T9 (Kruskal–Wallis, *p* < 0.001). The largest performance variation over the course of the day was 14.88% in T3 with predicted performance values of 80.83 ± 0.92% of maximum performance in the morning, 89.97 ± 0.41% in the afternoon, and 95.71 ± 0.50% in the evening (Kruskal–Wallis, *p* < 0.001) (Figures 3A–L).

**Figure 4:**
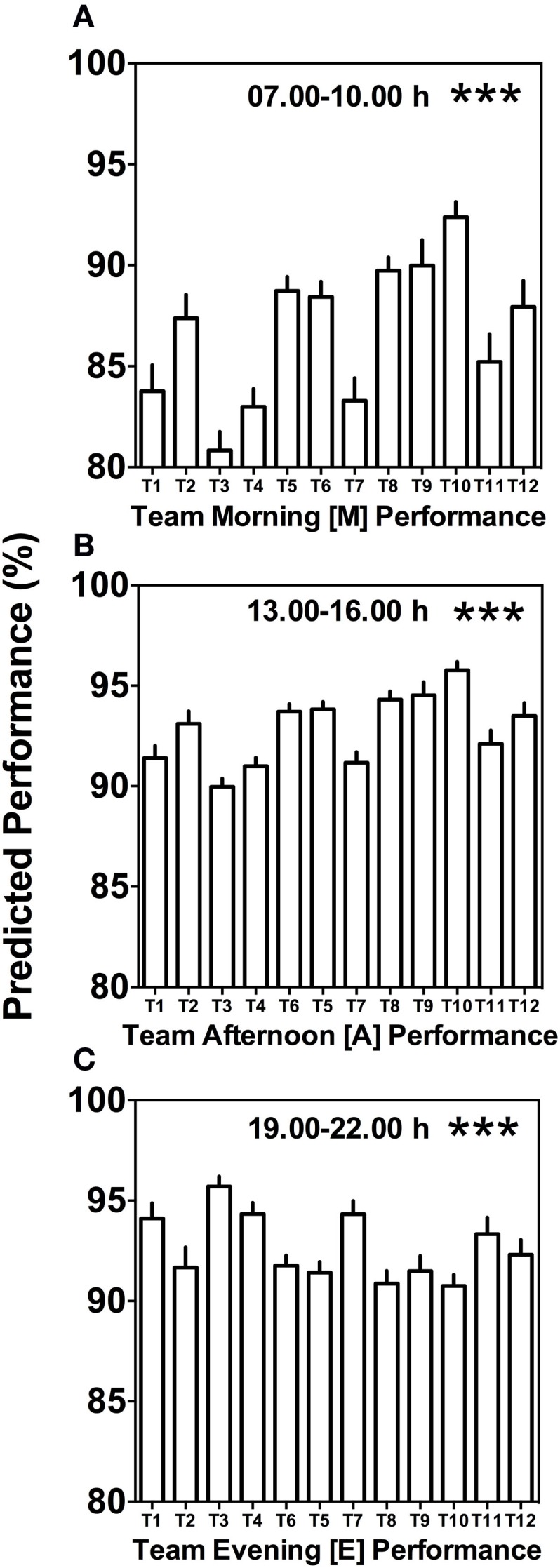
**Between-team performance differences are most pronounced in the morning and afternoon**. **(A)** Average predicted team performance in the morning ([*M*] = 07.00 and 10.00 h performance tests), **(B)** average predicted team performance in the afternoon ([*A*] = 13.00 and 16.00 h performance tests), and **(C)** average predicted team performance in the evening ([*E*] = 19.00 and 22.00 h performance tests). Bars represent mean values + SE of predicted team performance levels based on the composition of circadian phenotypes within each team. Statistical analysis was carried out using Kruskal–Wallis non-parametric tests combined with Dunn’s multiple comparison test. ***p < 0.001. Predicted performance represents percentage of maximum performance attained. Dunn’s multiple comparison test results are shown in Table S1 in Supplementary Material.

Morning (*M*), afternoon (*A*), and evening (*E*) comparison between teams predicts highly significant differences (Kruskal–Wallis, *p* < 0.001) with clear differences in variability between the different times of day (Figure 4). Kruskal–Wallis and Dunn’s post-test allowed 66 possible combinations of analyses between the teams; for both morning (*M*) and afternoon (*A*) performance, 20 out of the 66 possible team comparisons were significantly different, while for evening (*E*) performance, 12 out of 66 possible team comparisons differed significantly (Figures 4A–C; Table S1 in Supplementary Material).

### Impact of age and gender on predicted performance

**Figure 5:**
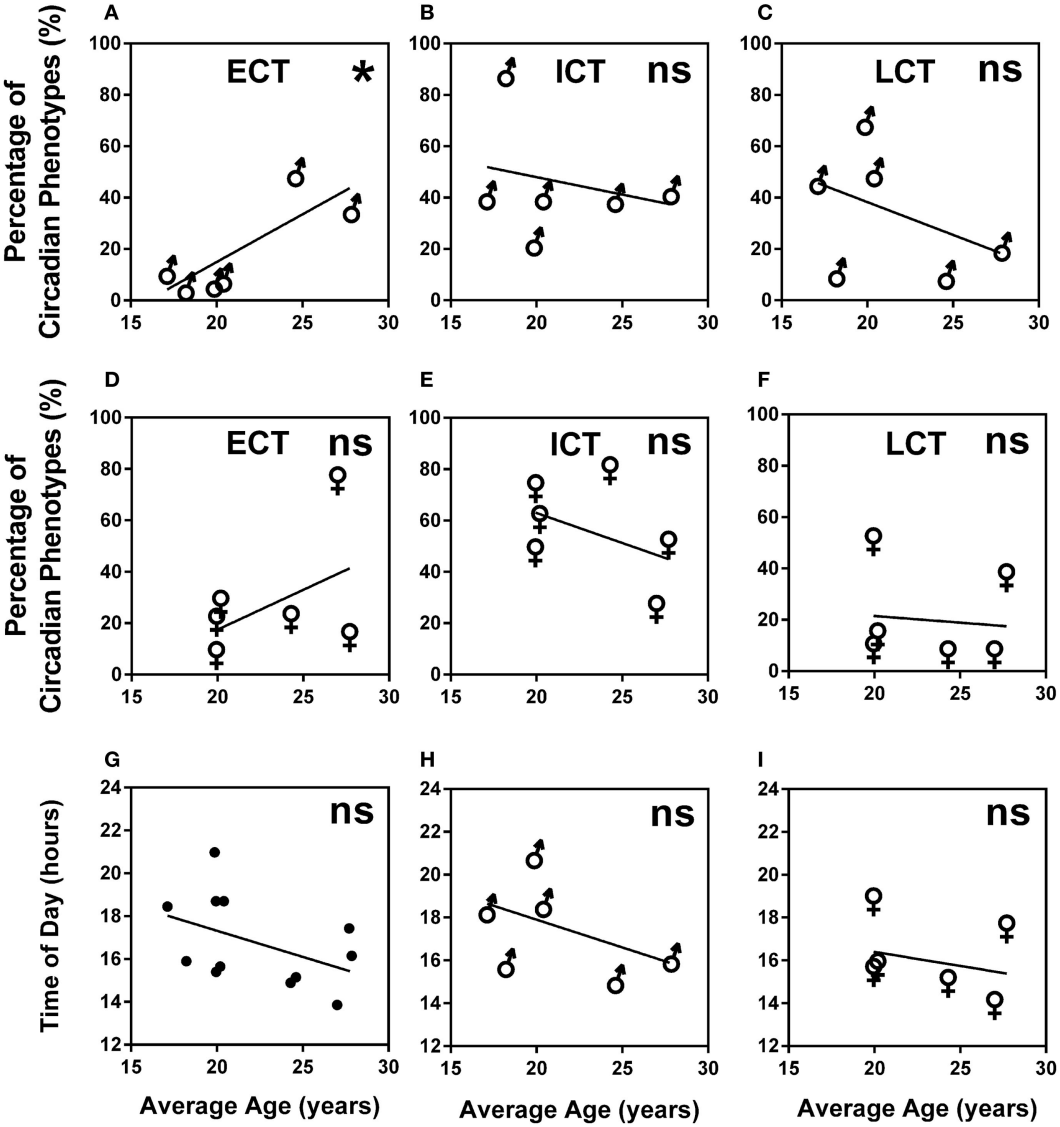
**Age and gender are negligible predictors of circadian phenotype composition and peak performance times**. **(A)** Male teams average age vs. percentage of ECTs. **(B)** Male teams average age vs. percentage of ICTs. **(C)** Male teams average age vs. percentage of LCTs. **(D)** Female teams average age vs. percentage of ECTs. **(E)** Female teams average age vs. percentage of ICTs. **(F)** Female teams average age vs. percentage of LCTs. **(G)** Age vs. predicted peak performance for all teams. **(H)** Age vs. predicted peak performance in male teams. **(I)** Age vs. predicted peak performance in female teams. Statistical analysis was carried out using linear regression analysis; ns, not significant, *p < 0.05. Early circadian phenotypes are labeled as ECT, intermediate circadian phenotypes as ICT and late circadian phenotypes as LCT; male teams, ♂; female teams, ♀.

A higher percentage of males were LCT (45%) as compared to females (24%), whilst the percentage of ECTs was similar for both males and females (12 and 17%, respectively). Average age for ECTs was 24.16 ± 0.97, for ICTs 21.36 ± 0.35, and for LCTs 20.51 ± 0.36 showing a significant increase of ECTs with age (Kruskal–Wallis, *p* < 0.001). Analysis of age vs. circadian phenotype composition within teams showed that the significant increase of ECTs with age was caused by a significant positive correlation between increasing age and increasing percentage of ECTs (*p* = 0.0239) in males only (Figures 5A–F). Age did not correlate with predicted peak performance times (Figures 5G–I).

### Self-reported mental and physical performance

**Figure 6:**
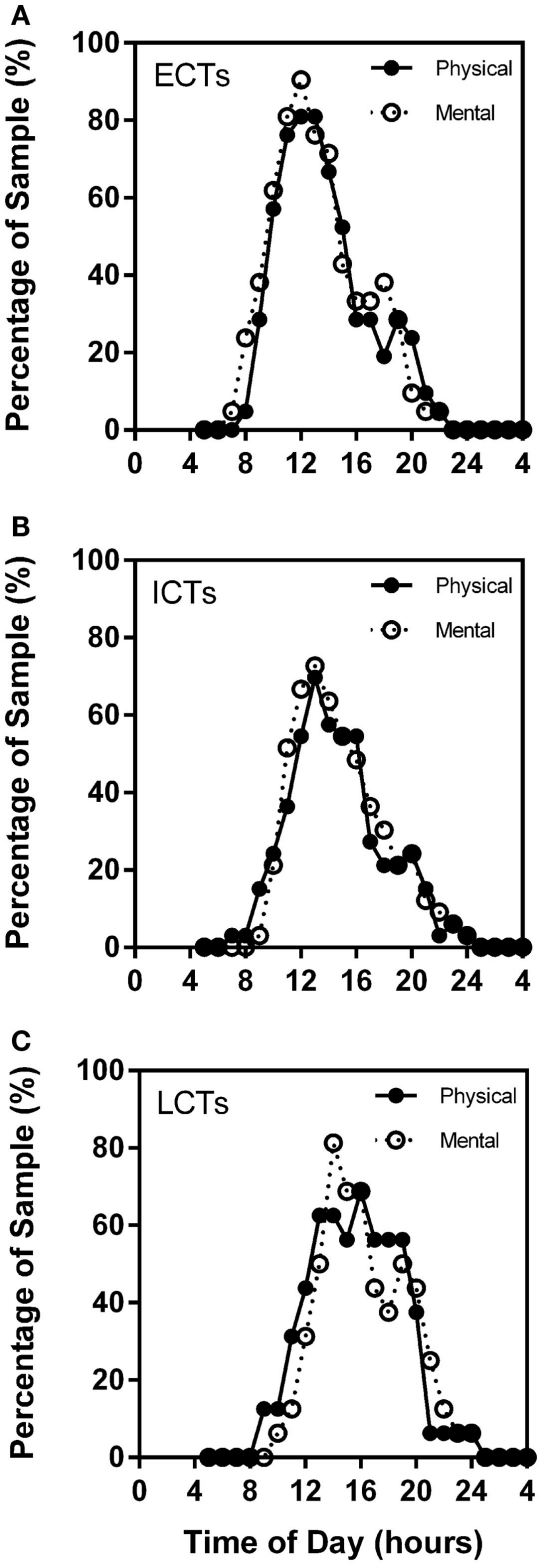
**Subjective mental and physical performance are strongly linked**. Frequency plots show both self-reported high mental and physical performance depending on time of day. Subjective physical performance is shown by the solid black line and subjective mental performance by the stippled line. *X*-axes = time of day in hours. *Y*-axes = percentage of total sample (%). **(A)** ECTs. **(B)** ICTs. **(C)** LCTs.

Through the analysis of the chronometric tests, self-reported high mental and physical activity frequency curves overlap considerably in each of the circadian phenotypes. Highest percentage of ECTs reported highest mental activity at *X* = 12 (90.48%) and highest physical performance at *X* = 12 (80.95%). ICT curves were similar with highest mental performance at *X* = 13 (72.73%) and physical at *X* = 13 (69.70%). LCT curves were delayed as compared to ECTs and ICTs with highest mental performance at *X* = 14 (81.25%) and highest physical at *X* = 16 (68.75%) (Figures 6A–C).

## Discussion

A detailed understanding of factors contributing to performance is a major goal for researchers, coaches, and managers in the sports world and the corporate world. Advances in technology have allowed detailed analysis of physiological and cognitive performance variables, such as heart rate (39, 40), distance covered (41–43), direction of runs (44, 45), body temperature (46), maximal oxygen uptake (47), and cognitive abilities (23, 48–52). When exploring requirements for appropriate performance evaluation in teams, strategy, coordination, psychology, and skills have to be considered as well as comprehensive performance indicators, both at the level of the individual and the team (53, 54).

Tools for evaluating team performance incorporate both cognitive and behavioral processes that individuals invest toward similar or shared goals. It is important that when analyzing team performance, both individual and team goals are considered (55). To optimize team effectiveness and performance, there is a need to understand individual and team learning. Individuals’ perception of reality has been shown to be a predictor of performance, also known as mental models (56). If roles, goals, and tactics are similar between individuals then team mental models could predict team performance. In competing sports teams, optimal performance as a team with each team member delivering their personal best performance and playing an equally important role in achieving a common goal is a general principle. Very recently, Dijkstra et al. suggested a health management and coaching model for the optimization of performance (57). Comparable to other theoretical approaches to performance optimization, this model does not consider individual variations of sleep/wake rhythms, circadian rhythms of physiology, or the relevance of time of day in training efficiency (26, 27, 58, 59), while disruptions of circadian rhythmicity can lead to sleep disorders, cognitive impairments, and ultimately have impact on both individual and team performance (60, 61). For example, traveling across time zones can have detrimental effects on both physical and mental performance (62–64). There has been increasing research on using chronobiological knowledge to readjust circadian misalignment, thereby improving performance, mood, and quality of sleep (61).

Our simulation of competition at different times of day (Figure 3) revealed T10 as the physically strongest team in the morning and afternoon but weakest team in the evening, while T3 was the strongest team in the evening and weakest team in the morning and afternoon. These data suggest that teams with a large proportion of late types are disadvantaged in morning competitions, while teams with either large proportions of early types or large proportions of intermediate types are disadvantaged in evening competitions. The strong link between physical performance and perceived mental performance may further contribute to these diurnal performance variations (20, 21).

With this paper, we establish the importance of circadian phenotype and individual diurnal performance variation as considerable performance indicators in groups of individuals or teams. Our results show that both, predicted performance levels as well as peak performance times in the course of a day, are influenced by the circadian phenotype distribution within a team suggesting that it is the number of late circadian phenotypes that has the strongest influence on peak performance of a team in the course of a day. Age and gender, however, are only weak predictors of circadian phenotype despite a higher percentage of early circadian phenotypes found in females and an increasing percentage of early phenotypes with increasing age in males. This supports previous studies that have reported more ECTs in females and older age groups and more LCTs in males and younger age groups (65–67); other studies, however, suggested that morningness/eveningness preference is largely independent of gender, indicating that it is a stable characteristic that may be better explained by endogenous factors (68), and that sleep disturbances between different chronotypes were not influenced by age or gender (69). Consistent with these reports, age did not correlate with the differences in peak performances in our study, while all performance parameters differed significantly with circadian phenotype distribution within teams. In our study, age did not have an effect on performance in the course of a day.

Circadian phenotype distribution within a team is not related to age and is not predictable; this is supported by our results showing an exclusively male team with an average age of 17.23 years and an exclusively female team with average age 27.71 years showing highly similar circadian phenotype composition and, as a consequence, very similar performance curves regardless of the age and gender differences (Figure 2, T1 and T11).

Our model of predicted performance shows, for the first time, how varied team performance can be, both mental and physical, in the course of a day depending on the composition of circadian phenotypes with a 7-h difference in peak performance times between the teams studied. Our novel tools, including a chronometric test specifically designed for the analysis of circadian phenotypes and performance analysis at different times of day, could help teams to have a “circadian advantage” over other teams due to detailed knowledge about peak performance and times and peak performance levels of individual team members and the team as a whole. The need to understand the biological clocks and to develop new strategies and techniques to enhance performance taking into account circadian influences has been shown in various studies (70–74). Knowledge into team compositions of circadian phenotypes could allow better preparation for demanding tasks, competitions for athletes, adjustment of work or training schedules, and more effective people management. For example, a selection of a higher number of players of the late circadian phenotype for evening matches, such as Champions league matches in football, could shift peak team performance to a later time of day and enhance team performance. Thus, knowledge about circadian phenotype has the potential to become one of the important criteria for team member recruitment depending on routines and schedules with afternoon tasks benefiting from a higher number of early and intermediate circadian phenotypes and evening tasks benefiting from a higher number of late circadian phenotypes. For the evaluation of team performance to be efficient, a considerable number of factors need to be taken into consideration, including team structure (75), individual and team goals (55, 76), individual and team mental models (56), feedback and reflection (77), motivation (78) as well as an understanding behind physical fitness (19), cognitive abilities (13, 14, 23, 24), and health (61, 79, 80). With this study, we provide new insight into the analysis and interpretation of team performance by introducing circadian phenotype as a relevant team performance indicator.

## Conflict of Interest Statement

The authors declare that the research was conducted in the absence of any commercial or financial relationships that could be construed as a potential conflict of interest.

## Supplementary Material

The Supplementary Material for this article can be found online at http://journal.frontiersin.org/article/10.3389/fneur.2015.00208

## Funding

This study was carried out as a MIBTP PhD mini-project supported by the Biotechnology and Biological Sciences Research Council (BBSRC), grant number BB/J014532/1.
