# Supplementary material for: Circadian Phenotype Composition is a Major Predictor of Diurnal Physical Performance in Teams
Source: Front Neurol. 2015 Oct 1;6:208. doi: 10.3389/fneur.2015.00208 (PMC4589674; doi:10.3389/fneur.2015.00208)
Supplement: Supplementary file 1 [file Table_1.DOCX]

| **Morning**  **[M]** | | **Afternoon**  **[A]** | | **Evening**  **[E]** | |
| --- | --- | --- | --- | --- | --- |
| T1 vs T2 | ns | T1 vs T2 | ns | T1 vs T2 | ns |
| T1 vs T3 | ns | T1 vs T3 | ns | T1 vs T3 | ns |
| T1 vs T4 | ns | T1 vs T4 | ns | T1 vs T4 | ns |
| T1 vs T5 | ns | T1 vs T5 | ns | T1 vs T5 | ns |
| T1 vs T6 | ns | T1 vs T6 | ns | T1 vs T6 | ns |
| T1 vs T7 | ns | T1 vs T7 | ns | T1 vs T7 | ns |
| T1 vs T8 | ns | T1 vs T8 | ns | T1 vs T8 | ns |
| **T1 vs T9** | ***** | **T1 vs T9** | ***** | T1 vs T9 | ns |
| **T1 vs T10** | ******* | **T1 vs T10** | ******* | T1 vs T10 | ns |
| T1 vs T11 | ns | T1 vs T11 | ns | T1 vs T11 | ns |
| T1 vs T12 | ns | T1 vs T12 | ns | T1 vs T12 | ns |
| T2 vs T3 | ns | T2 vs T3 | ns | **T2 vs T3** | ****** |
| T2 vs T4 | ns | T2 vs T4 | ns | T2 vs T4 | ns |
| T2 vs T5 | ns | T2 vs T5 | ns | T2 vs T5 | ns |
| T2 vs T6 | ns | T2 vs T6 | ns | T2 vs T6 | ns |
| T2 vs T7 | ns | T2 vs T7 | ns | T2 vs T7 | ns |
| T2 vs T8 | ns | T2 vs T8 | ns | T2 vs T8 | ns |
| T2 vs T9 | ns | T2 vs T9 | ns | T2 vs T9 | ns |
| T2 vs T10 | ns | T2 vs T10 | ns | T2 vs T10 | ns |
| T2 vs T11 | ns | T2 vs T11 | ns | T2 vs T11 | ns |
| T2 vs T12 | ns | T2 vs T12 | ns | T2 vs T12 | ns |
| T3 vs T4 | ns | T3 vs T4 | ns | T3 vs T4 | ns |
| **T3 vs T5** | ******* | **T3 vs T5** | ******* | **T3 vs T5** | ******* |
| **T3 vs T6** | ******* | **T3 vs T6** | ******* | **T3 vs T6** | ******* |
| T3 vs T7 | ns | T3 vs T7 | ns | T3 vs T7 | ns |
| **T3 vs T8** | ******* | **T3 vs T8** | ******* | **T3 vs T8** | ******* |
| **T3 vs T9** | ******* | **T3 vs T9** | ******* | **T3 vs T9** | ****** |
| **T3 vs T10** | ******* | **T3 vs T10** | ******* | **T3 vs T10** | ******* |
| T3 vs T11 | ns | T3 vs T11 | ns | T3 vs T11 | ns |
| **T3 vs T12** | ******* | **T3 vs T12** | ******* | **T3 vs T12** | ***** |
| **T4 vs T5** | ****** | **T4 vs T5** | ****** | **T4 vs T5** | ****** |
| **T4 vs T6** | ******* | **T4 vs T6** | ******* | **T4 vs T6** | ***** |
| T4 vs T7 | ns | T4 vs T7 | ns | T4 vs T7 | ns |
| **T4 vs T8** | ****** | **T4 vs T8** | ****** | **T4 vs T8** | ****** |
| **T4 vs T9** | ******* | **T4 vs T9** | ******* | T4 vs T9 | ns |
| **T4 vs T10** | ******* | **T4 vs T10** | ******* | T4 vs T10 | ns |
| T4 vs T11 | ns | T4 vs T11 | ns | T4 vs T11 | ns |
| **T4 vs T12** | ***** | **T4 vs T12** | ***** | T4 vs T12 | ns |
| T5 vs T6 | ns | T5 vs T6 | ns | T5 vs T6 | ns |
| **T5 vs T7** | ***** | **T5 vs T7** | ***** | **T5 vs T7** | ***** |
| T5 vs T8 | ns | T5 vs T8 | ns | T5 vs T8 | ns |
| T5 vs T9 | ns | T5 vs T9 | ns | T5 vs T9 | ns |
| T5 vs T10 | ns | T5 vs T10 | ns | T5 vs T10 | ns |
| T5 vs T11 | ns | T5 vs T11 | ns | T5 vs T11 | ns |
| T5 vs T12 | ns | T5 vs T12 | ns | T5 vs T12 | ns |
| **T6 vs T7** | ***** | **T6 vs T7** | ***** | T6 vs T7 | ns |
| T6 vs T8 | ns | T6 vs T8 | ns | T6 vs T8 | ns |
| T6 vs T9 | ns | T6 vs T9 | ns | T6 vs T9 | ns |
| T6 vs T10 | ns | T6 vs T10 | ns | T6 vs T10 | ns |
| T6 vs T11 | ns | T6 vs T11 | ns | T6 vs T11 | ns |
| T6 vs T12 | ns | T6 vs T12 | ns | T6 vs T12 | ns |
| **T7 vs T8** | ***** | **T7 vs T8** | ***** | **T7 vs T8** | ***** |
| **T7 vs T9** | ****** | **T7 vs T9** | ****** | T7 vs T9 | ns |
| **T7 vs T10** | ******* | **T7 vs T10** | ******* | T7 vs T10 | ns |
| T7 vs T11 | ns | T7 vs T11 | ns | T7 vs T11 | ns |
| T7 vs T12 | ns | T7 vs T12 | ns | T7 vs T12 | ns |
| T8 vs T9 | ns | T8 vs T9 | ns | T8 vs T9 | ns |
| T8 vs T10 | ns | T8 vs T10 | ns | T8 vs T10 | ns |
| T8 vs T11 | ns | T8 vs T11 | ns | T8 vs T11 | ns |
| T8 vs T12 | ns | T8 vs T12 | ns | T8 vs T12 | ns |
| T9 vs T10 | ns | T9 vs T10 | ns | T9 vs T10 | ns |
| T9 vs T11 | ns | T9 vs T11 | ns | T9 vs T11 | ns |
| T9 vs T12 | ns | T9 vs T12 | ns | T9 vs T12 | ns |
| **T10 vs T11** | ****** | **T10 vs T11** | ****** | T10 vs T11 | ns |
| T10 vs T12 | ns | T10 vs T12 | ns | T10 vs T12 | ns |
| T11 vs T12 | ns | T11 vs T12 | ns | T11 vs T12 | ns |

**Supplementary table 1. Dunn’s multiple comparison test results for the comparison of performance between teams during morning (M), afternoon (A) and evening (E) as shown in figure 4.** Teams are labelled T1 to T12. Statistical analysis was carried out using Kruskal Wallis test combined with Dunn’s multiple comparison test. ns=not significant, p<0.05=*, p<0.01=**, p<0.001=***.
